# Virus Control of Cell Metabolism for Replication and Evasion of Host Immune Responses

**DOI:** 10.3389/fcimb.2019.00095

**Published:** 2019-04-18

**Authors:** María Maximina B. Moreno-Altamirano, Simon E. Kolstoe, Francisco Javier Sánchez-García

**Affiliations:** ^1^Laboratorio de Inmunorregulación, Departamento de Inmunología, Escuela Nacional de Ciencias Biológicas, Instituto Politécnico Nacional, Mexico City, Mexico; ^2^School of Health Sciences, University of Portsmouth, Portsmouth, United Kingdom

**Keywords:** viruses, cell metabolism, mitochondria, immune response, viral evasion

## Abstract

Over the last decade, there has been significant advances in the understanding of the cross-talk between metabolism and immune responses. It is now evident that immune cell effector function strongly depends on the metabolic pathway in which cells are engaged in at a particular point in time, the activation conditions, and the cell microenvironment. It is also clear that some metabolic intermediates have signaling as well as effector properties and, hence, topics such as immunometabolism, metabolic reprograming, and metabolic symbiosis (among others) have emerged. Viruses completely rely on their host's cell energy and molecular machinery to enter, multiply, and exit for a new round of infection. This review explores how viruses mimic, exploit or interfere with host cell metabolic pathways and how, in doing so, they may evade immune responses. It offers a brief outline of key metabolic pathways, mitochondrial function and metabolism-related signaling pathways, followed by examples of the mechanisms by which several viral proteins regulate host cell metabolic activity.

## Introduction

**Figure 1:**
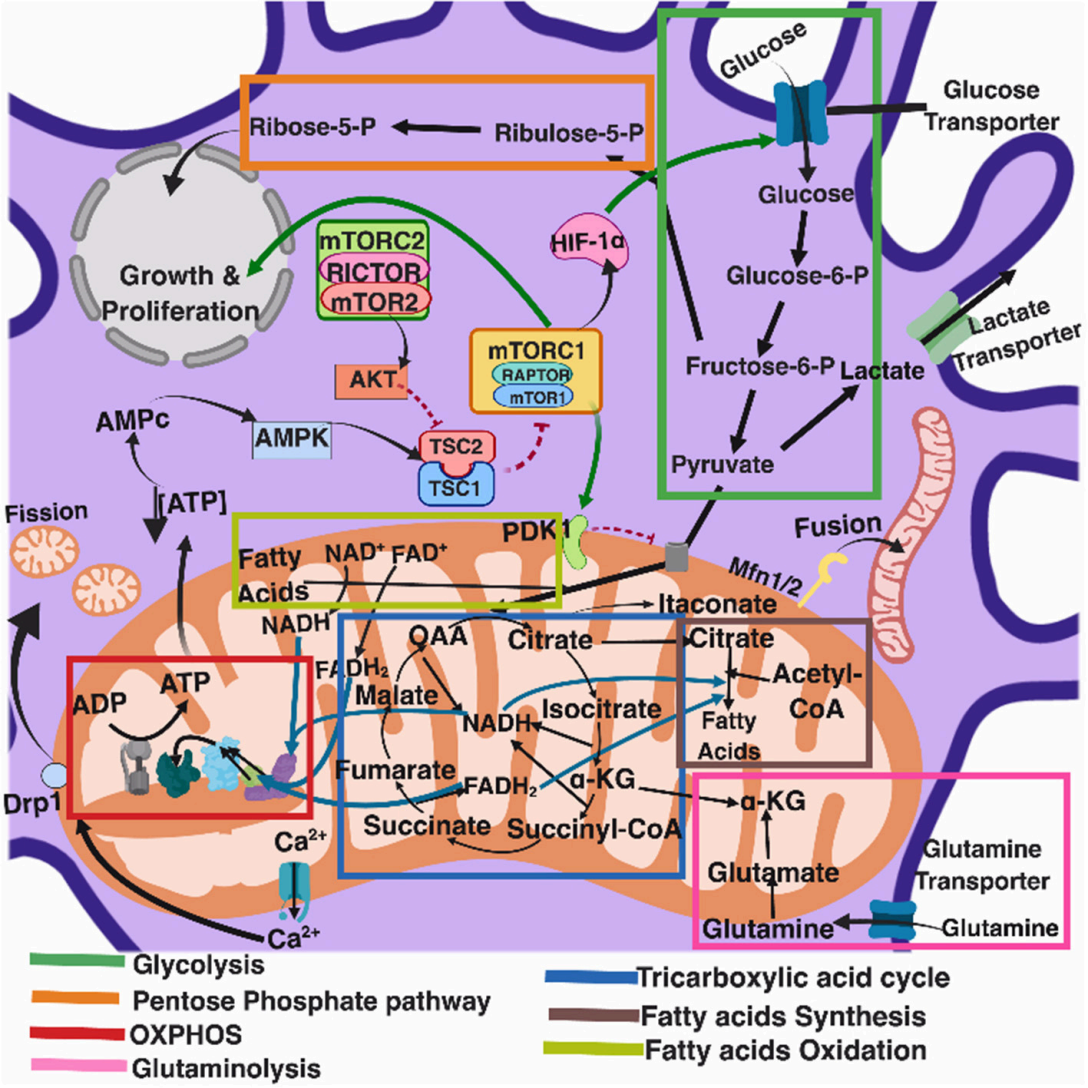
Eukaryotic cell metabolism. Bioenergetic and biosynthetic pathways interconnect glycolysis, glutaminolysis, PPP (pentose phospahate pathway), TCA (tricarboxylic acid cycle), FAO (fatty acid oxidation), fatty acid synthesis, aminoacid synthesis, metabolic sensors such as the AMPK, mTORC1, and mTORC2 pathways, and are also dependent on calcium homeostasis, mitochondrial membrane potential and mitochondrial dynamics. All together they influence cell function and may be the targets of several viruses.

Several recent comprehensive reviews have highlighted the key role of eukaryotic cell metabolism in immunity (Ganeshan and Chawla, 2014; O'Neill and Pearce, 2016; O'Neill et al., 2016). Six main and interconnected metabolic pathways have a role in the immune response: glycolysis; the pentose phosphate pathway (PPP); the tricarboxylic acid cycle (TCA), also known as Krebs cycle; the fatty acid oxidation (FAO), also known as β-oxidation; as well as the fatty acid and amino acid synthesis pathways (Figure 1).

Mitochondria take central stage in cellular metabolism since TCA, FAO, oxidative phosphorylation (OXPHOS), calcium buffering, and heme biosynthesis take place within this organelle (Mishra and Chan, 2016).

Energetic and biosynthetic metabolism is fueled by carbon sources, including glucose and glutamine (DeBerardinis and Cheng, 2010), which are taken up by the cells by glucose and glutamine transporters, respectively (Bhutia and Ganapathy, 2016; Navale and Paranjape, 2016).

Once in the cytosol, glucose is converted to pyruvate, via glycolysis, yielding two molecules of ATP and two molecules of NADH (which acts as a cofactor in several enzymatic reactions) per unit of glucose. The glycolysis pathway is also the source of biosynthetic intermediates that serve the purpose of ribose and nucleotides synthesis (glucose-6-phosphate into ribulose 5-phosphate), amino acids (3-phosphoglycerate enters the serine biosynthetic pathway), and fatty acids (by the sequential conversion of glycolysis-derived pyruvate into the TCA intermediate citrate that may be exported from the mitochondria to the cytosol, where it is converted into acetyl-coA).

Glycolysis-derived pyruvate is either converted to lactate, which is exported out of the cells, or converted into acetyl-CoA that enters the TCA cycle through the aldol condensation with oxaloacetate to form citrate (O'Neill et al., 2016). Citrate is then sequentially converted to isocitrate, α-ketoglutarate, succinyl CoA, succinate, fumarate, malate, and oxaloacetate, which starts a new round of the TCA cycle by its reaction with pyruvate-derived acetyl CoA. Fatty acids can also be converted into acetyl CoA through FAO, linking this metabolic pathway with the TCA cycle. Two major products of both the TCA cycle and FAO are NADH and FADH2, which can transfer electrons to the mitochondrial electron transport chain coupled with OXPHOS and the generation of ATP (O'Neill et al., 2016). In addition, succinate, an intermediate in the TCA cycle, is also an electron donor for the mitochondrial respiratory chain at complex II (succinate dehydrogenase) (Rich and Maréchal, 2010).

The pentose phosphate pathway involves a non-oxidative as well as an oxidative branch; the first allows for the diversion from glycolysis intermediates toward the synthesis of nucleotide and amino acid precursors, while the second generates reducing equivalents of nicotinamide adenine dinucleotide phosphate hydrogen (NADPH), which maintain a favorable cellular redox environment and allows fatty acid synthesis (O'Neill et al., 2016).

Fatty acid synthesis uses glycolysis, TCA cycle, and pentose phosphate pathway metabolic intermediates. TCA cycle-derived citrate may be exported from the mitochondria to the cytosol and then ATP citrate lyase converts citrate to acetyl-coA, which in turn may be carboxylated by acetyl-CoA carboxylase (ACC) producing malonyl-CoA. Furthermore, fatty acid synthase and NADPH elongate fatty acid chains (O'Neill et al., 2016).

Glutamine is also a primary source of energy as it is converted to glutamate and then to α-ketoglutarate, which enters the TCA cycle (DeBerardinis and Cheng, 2010).

Immune system cells preferentially follow one or other metabolic pathway, depending on cell type, differentiation status, activation conditions, and microenvironment. Resting T lymphocytes rely mostly on OXPHOS, whereas activated and proliferating T lymphocytes upregulate the expression of the glucose transporter glut-1 and key glycolytic enzymes, relying mostly on glycolysis (Frauwirth et al., 2002; Pearce and Pearce, 2013).

Memory T lymphocytes use OXPHOS (Pearce and Pearce, 2013), “classically activated” macrophages (stimulated with LPS plus IFN-γ)—also referred to as M1 macrophages—engage in glycolysis, whereas alternatively activated macrophages (stimulated with IL-4)—also referred to as M2 macrophages—use OXPHOS and FAO to generate energy (Rodríguez-Prados et al., 2010). Stimulated macrophages and dendritic cells engage in glycolysis after activation through pattern recognition receptors (PRRs) (O'Neill and Pearce, 2016).

Neutrophils rely mostly on glycolysis (Pearce and Pearce, 2013) and the release of neutrophil extracellular traps (NETs) is dependent on the increase in cell membrane glut-1, glucose uptake, and the glycolytic rate (Rodriguez-Espinosa et al., 2015).

Activated B lymphocytes undergo metabolic reprogramming in response to changing energetic and biosynthetic demands, and long-lived plasma cells uptake glucose and glutamine at a higher rate; glucose is used to generate pyruvate for spare respiratory capacity, and glutamine is used as a carbon source for mitochondrial anaplerotic reactions and respiration, promoting cell survival (Jellusova and Rickert, 2016; Lam et al., 2018).

Switching metabolic pathways (metabolic reprograming) leads to changes in cell function (Buck et al., 2017) and the metabolic microenvironment, i.e., tissue O_2_ tension, or the concentration of metabolites such as lactate determines cell immune responses (Romero-Garcia et al., 2016).

Interestingly, viral infections such as ocular infection with herpes simplex virus-1 (HSV-1) may change blood glucose levels in the course of infection (Varanasi et al., 2017). Moreover, if glucose utilization is pharmacologically limited *in vivo* in the inflammatory phase, lesions diminish but, if glucose utilization is limited in the acute phase of infection when the replicating virus is still present in the eye, infected mice become susceptible to the lethal effects of HSV-1 infection as the virus spreads to the brain, causing encephalitis (Varanasi et al., 2017). This highlights the fundamental relationship between cell metabolism, immune response, and viral pathogenesis.

## Anti-Viral Immune Responses

**Figure 2:**
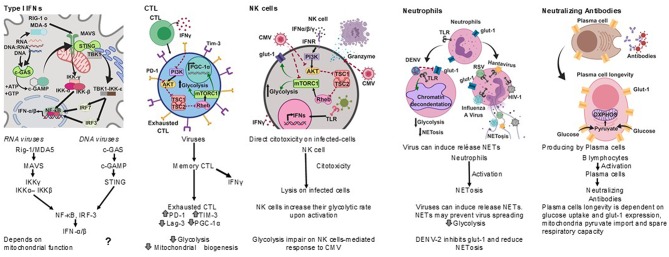
Antiviral immune responses. Type I interferons, cell cytotoxicity, neutrophil extracellular traps and neutralizing antibodies protect against viral infections, each type of response has a metabolic hallmark. Viruses may target specific metabolic pathways for immune evasion.^?^, not known.

Among the most effective antiviral immune responses is the production of several type I interferons (Figure 2); interferon-α (IFN-α) subtypes and interferon-β (IFN-β), which along with IFN-ε, IFN-τ, IFN-κ, IFN-ω, IFN-δ, and IFN-ζ, are collectively referred to as type I interferons; most cells can produce IFN-α and IFN-β following cell activation through the recognition of viral nucleic acids (McNab et al., 2015).

The RIG-I-MDA5-mitochondrial antiviral-signaling protein (MAVS) axis is the major sensing pathway for RNA viruses, while the axis composed of the cyclic guanosine monophosphate (cGMP)-adenosine monophosphate (AMP) synthase (cGAS) and the stimulator of interferon genes (STING) is the major sensing pathway for DNA viruses (Wu and Chen, 2014). However, there is recent evidence that the cGAS-STING pathway may also restrict the infection by RNA viruses, thus suggesting a connection between the sensing of cytosolic DNA and RNA (Ni et al., 2018).

Both anti-viral pathways converge in the activation of two main transcription factors that regulate the expression of type-I interferons, nuclear factor kappa B (NFκB) and interferon regulatory factor 3 (IRF3). In the case of the RIG-I-MDA5-MAVS pathway, their activation depends on mitochondrial function (Seth et al., 2005; Koshiba, 2013).

Both IFN-α and IFN-β activate the expression of interferon-stimulated genes (ISGs) through the Janus kinase-signal transducer and activator of transcription (JAK-STAT) signaling pathway, leading to the inhibition of viral replication and assembly (Darnell et al., 1994; Seth et al., 2005).

Cytotoxic T lymphocytes (CTL) play an important role in the clearance of viral infections (Figure 2); memory CTL can be activated by low concentrations of antigen, readily producing cytokines and the lysis of infected cells, thus preventing dissemination (Veiga-Fernandes et al., 2000).

Upon acute viral infection, virus-specific memory CTL quickly produce IFN-γ. However, around 18 h after infection the number of IFN-γ producing CTL drops concomitantly with the upregulation of inhibitory receptors. It has been suggested that the decrease in the synthesis of IFN-γ by CTL is an active regulatory process (Hosking et al., 2013) reminiscent of T cell exhaustion, a process also known to take place during chronic viral infections (Yi et al., 2010; Wherry, 2011).

A hallmark of T cell exhaustion is the upregulation of inhibitory receptors such as programmed death-1 (PD-1), T cell immunoglobulin mucin-3 (Tim-3), and lymphocyte activation gene-3 (Lag-3) (Freeman et al., 2000; Barber et al., 2006). Interestingly, PD-1 negatively regulates glycolysis, represses the transcriptional co-activator peroxisome proliferator-activated receptor-gamma co-activator (PGC)-1alpha (PGC-1α), which plays an important role in the regulation of carbohydrate and lipid metabolism, and impairs CTL responses (Bengsch et al., 2016).

Other anti-viral cell-mediated immune responses include NK cell cytotoxicity (Hammer et al., 2018) and neutrophil extracellular traps (NETs) (Schönrich and Raftery, 2016) (Figure 2).

Natural killer (NK) cells have anti-viral activities as they exert direct cytotoxicity on virus infected-cells, and readily produce IFN-γ. NK cells increase their glycolytic rate upon activation (Gardiner and Finlay, 2017), and disruption of glycolysis impairs NK cell-mediated responses to Cytomegalovirus (CMV), for instance (Mah et al., 2017).

Neutrophils are considered a first line of defense against pathogens. However, their role in the control of viral infections is not as clear as for other pathogens (Galani and Andreakos, 2015). It has recently been recognized that viruses can induce the release of neutrophil extracellular traps (NETs), and the mechanisms by which NETs could contribute to anti-viral immunity are emerging (Hammer et al., 2018).

Several viruses, including Hantaan virus (HTNV), H1N1 Influenza A virus (IAV), human immunodeficiency virus (HIV-1), and Respiratory Syncytial virus (RSV), directly stimulate neutrophils to release NETs (Raftery et al., 2014; Delgado-Rizo et al., 2017), and both IFN-α and IFN-γ can prime mature neutrophils to release NETs upon further stimulation (Martinelli et al., 2004; Hammer et al., 2018).

HIV-1 may also prevent the release of NETs by inducing dendritic cells to produce IL-10, which in turn suppresses the reactive oxygen species (ROS)-dependent release of NETs (Saitoh et al., 2012; Hammer et al., 2018).

The Dengue virus serotype-2 (DENV-2) down-modulates the phorbol 12- myristate 13- acetate-(PMA) induced release of NETs, and it has been proposed that one of the mechanisms for this is the interference with the mobilization of the glucose transporter glut-1 to the cell membrane and consequently with the glucose uptake (Moreno-Altamirano et al., 2015).

NETs may prevent virus spreading by being trapped by electrostatic attraction or be inactivated by molecules associated with NETs, such as myeloperoxidase and α-defensins (Saitoh et al., 2012; Hammer et al., 2018).

Antibodies are also important anti-viral effectors (Figure 2), and whereas cytotoxic lymphocytes can eliminate infected cells, antibodies are capable of both eliminating infected cells and neutralizing viruses, thereby preventing cell infection. The production of protective antibodies over prolonged periods constitutes a first line of defense against reinfection and, therefore, survival of antibody-producing plasma cells is determinant (Dörner and Radbruch, 2007). It is now known that plasma cell longevity is dependent on enhanced glut-1 expression and glucose uptake, mitochondria pyruvate import and spare respiratory capacity, and that nutrient uptake and catabolism distinguish plasma cell subsets with different lifespans and rates of secreted antibodies (Lam et al., 2016, 2018).

Taken together, it emerges that the activity of immune system cells is dependent on cell metabolism and that viruses could target cell metabolism to evade anti-viral immune responses. The next sections explore some specific mechanisms by which viruses may interfere with cell metabolism.

## Mitochondrial Anti-Viral Signaling (MAVS) and Virus Subversion of MAVS

Mitochondria constitute a metabolic hub, so if a virus is to take control of host metabolism, targeting mitochondria is perhaps the best way.

In 2005 Seth et al. reported the identification of a new protein essential for the activation of the transcription factors NFκB and IRF3 by RNA viruses. They named the protein MAVS and showed that this contains a C-terminal transmembrane domain that targets the mitochondrial outer membrane. Strikingly, they found that this transmembrane domain and the targeting to mitochondria are essential for MAVS signaling, opening a new avenue of research in which mitochondria took center stage in antiviral immunity (Seth et al., 2005).

In non-stimulated cells, NFκB is located in the cytoplasm, associated with its inhibitor IκBα. Upon stimulation with viruses, other pathogens or cytokines, the IκB kinase (IKK) is activated, leading to the phosphorylation of IκBα and its subsequent ubiquitination and proteasomal degradation. NFκB is then released and translocated to the nucleus, where it activates immune and inflammatory genes (Silverman and Maniatis, 2001; Seth et al., 2005).

IRF3 is located in the cytoplasm of non-stimulated cells, and following viral or other pathogen infection it becomes phosphorylated by TANK-binding kinase 1 (TBK1) and IKK kinases, allowing the formation of homodimers that can translocate into the nucleus and activate the synthesis of IFN-β, acting in synergy with NFκB (Yoneyama et al., 2002; Fitzgerald et al., 2003; Hiscott et al., 2003; Seth et al., 2005).

IRF7 can also be phosphorylated by TBK1 and IKK (tenOever et al., 2004), leading to the production of interferon-α (Honda et al., 2005; Seth et al., 2005). NFκB and IRFs are activated by RNA viruses as well as by other pathogens.

The entry of RNA viruses to the cells produces double-stranded RNA intermediates, which can be recognized by host cell pathogen recognition receptors (PRRs) including TLR -3, -7, -8, and -9 (Akira and Takeda, 2004; Seth et al., 2005).

The receptor Retinoic Acid-Induced Gene I (RIG-1) recognizes intracellular dsRNA and the interaction of viral RNA with RIG-1 leads to a change in its conformation, which then activates NFκB and IRF3 (Yoneyama et al., 2002; Sumpter et al., 2005).

The melanoma differentiation-associated gene 5 (MDA5) is a RIG-I-like protein involved in dsRNA signaling and apoptosis (Kovacsovics et al., 2002; Seth et al., 2005).

In 2011, Koshiba (Koshiba, 2013) demonstrated that mitochondrial fusion and mitochondrial membrane potential (Δψ_m_) are required for MAVS-mediated signaling. They showed that the deletion-targeting of mitofusin 1 (Mfn1) and mitofusin 2 (Mfn2), two molecules involved in mitochondrial fusion, prevented cells from producing interferons and pro-inflammatory cytokines in response to viral infection. This resulted in increased viral replication along with a reduced Δψ_m_, correlating with a reduced antiviral response. Interestingly, the reduction in Δψ_m_ did not affect the activation of IRF3, which acts downstream of MAVS, suggesting that Δψ_m_ and MAVS are coupled at the same stage in the RIG-1-like Receptor (RLR) signaling pathway (Koshiba, 2013).

In addition to mitochondria, MAVS are also found in peroxisomes and mitochondrial-associated membranes (MAMs) (Seth et al., 2005; Vazquez and Horner, 2015).

A natural target for the subversion of IFN type I-mediated antiviral response is the MAVS protein (Table 1). As an example, the influenza A virus encodes a protein called PB1-F2, which inhibits the induction of type I interferon at the level of the MAVS (Varga et al., 2012). PB1-F2 is an 87–90-amino-acid-long protein with a serine at position 66 (66S), which accounted for the virulence of the Spanish and avian flu pandemic viruses (H1N1 and H5N1, respectively). Interestingly, PB1-F2 66S has a higher affinity for MAVS than PB1-F2 66N, and more efficiently affects the Δψ_m_ than the wild-type PB1-F2 (Conenello et al., 2007).

**Table 1:** Viruses that subvert MAVS.

**Virus**	**Viral proteins**	**Effect**	**References**
Influenza A virus (IAV)	PB1-F2	Inhibition of type I IFN at the level of MAVS	Conenello et al., 2007
Influenza A virus H1N1(1918) and H5N1	PB1-F2 66S, PB1-F2 66N	Disruption of mitochondrial membrane potential and type I IFN response	Conenello et al., 2007
Hepatitis C virus (HCV)	NS3/4A	Inhibition of type I IFN response by cleaving of MAVS	Meylan et al., 2005

Other viruses, such as hepatitis C virus (HCV), induce the cleavage of MAVS from the outer membrane of mitochondria, reducing the interferon-producing response. In this case, the NS3/4A protein cleaves MAVS at cysteine 508 (Meylan et al., 2005; Bender et al., 2015; Vazquez and Horner, 2015).

Another family of pattern recognition receptors contain a nucleotide-binding and oligomerization domain (NOD) and is called the NLR (NOD-like receptor) family. NOD2 facilitates the activation of IRF3 and the synthesis of type I IFN in response to single-stranded RNA. Interestingly, the activation of NOD2 is dependent on MAVS (Sabbah et al., 2009; Moreira and Zamboni, 2012).

Recently, NLRX1 (also known as NOD5, NOD9, or NOD26), a member of the NLR family that localizes to the outer mitochondrial membrane, was shown to mediate MAVS degradation, allowing HCV to evade type I IFN-mediated antiviral response (Qin et al., 2017).

## cGAS-STING Anti-Viral Pathway and Its Subversion by Viruses

The cyclic guanosine monophosphate (cGMP)-adenosine monophosphate (AMP) synthase (cGAS) recognizes viral as well as bacterial double-stranded DNA (dsDNA) (Wu and Chen, 2014; Ni et al., 2018). After binding to dsDNA, cGAS catalyzes the synthesis of the second messenger cyclic guanosine monophosphate-adenosine monophosphate (cGAMP), which then binds to the stimulator of interferon genes (STING) adaptor protein on the endoplasmic reticulum (ER); STING, as a dimer, translocates from the endoplasmic reticulum to the Golgi complex, where it recruits TANK-binding kinase 1 (TBK1) which activates the transcription factors NFκB and IRF3, both of which translocate to the nucleus and induce the synthesis of type I interferons (Barber, 2015; Ni et al., 2018).

While the activation of the RIG-1-MDA5-MAVS antiviral signaling pathway clearly requires mitochondrial activity, in the form of mitochondrial dynamics and Δψ_m_, a metabolism-related component in the cGAS-STING antiviral signaling pathway has not been explicitly identified. However, several lines of research suggest crosstalk between cGAS-STING and metabolism. Firstly, the ER has been regarded as a separate metabolic compartment on the basis that the ER luminal micro-environment is different from the cytosol, that it contains its own pool of pyridine nucleotides, and that several metabolic pathways related to carbohydrate and steroid metabolism, biotransformation, and protein processing take place in the ER (Csalaa et al., 2006); viral infections may lead to ER stress and to the unfolded protein response (UPR) (Zhang and Wang, 2012); and the mitochondrial function in cells undergoing ER stress is compromised, particularly at the level of mitochondrial membrane potential, oxygen consumption, and ATP production (Wang et al., 2011). The ER stress and UPR synergy with the cGAS-STING antiviral signaling pathway still needs to be fully elucidated (Smith, 2014).

Among the DNA viruses that activate the cGAS-STING pathway are herpes simplex virus 1 (HSV-1), vaccinia virus (VV), and murine gamma herpesvirus 68 (MHV68). Interestingly, RNA viruses such as HIV-1 generate RNA: DNA hybrids as well as dsDNA that may activate the cGAS-STING pathway (Ma and Damania, 2016; Ni et al., 2018).

Of note, dengue virus (DENV)-induced mitochondrial damage leads to mitochondrial DNA release to the cytosol, and the activation of the cGAS-STING pathway (Sun et al., 2007). Since other viruses may cause mitochondrial damage (see below) it is plausible that other RNA viruses may activate cGAS-STING through mitochondrial DNA release.

Several DNA virus-associated proteins are known to interfere with the cGAS-STING pathway, as reviewed by Ni et al. (2018), either by interfering with DNA binding to cGAS, as is the case of Kaposi's sarcoma-associated herpesvirus (KSHV), Epstein Barr virus (EBV), and murine gammaherpesvirus-68 (MHV68,γHV68) tegument protein open reading frame 52 (ORF52), and the KSHV latency-associated nuclear antigen (LANA) protein which interact with cGAS (Wu et al., 2015; Zhang et al., 2016), or by targeting STING, as is the case for the HSV-1-infected cell protein 27 (ICP27) and the UL46 protein, the KSHV viral interferon regulatory factor 1 (vIRF1), the human papillomavirus 18 (HPV18) E7 oncoprotein, the human adenovirus 5 (hAd5) E1A oncoprotein, and the Hepatitis B virus (HBV) polymerase (Lau et al., 2015; Liu et al., 2015; Ma et al., 2015; Christensen et al., 2016;Deschamps and Kalamvoki, 2017).

A more recent development in the field is the finding that some RNA viruses are also capable to interfere with the cGAS-STING pathway, subverting its anti-viral effect (Ni et al., 2018).

Finally, it has been shown that single- or double-stranded DNA may attenuate glucose metabolism, leading to ATP depletion and so constitute a metabolic barrier for viral replication. However, the mechanism seems to be dependent on the activation of adenosine monophosphate (AMP)-activated protein kinase (AMPK) and the activation of mechanistic target of rapamycin complex 1 (mTORC1) (see below), but independent of the cGAS-STING anti-viral pathway (Zheng et al., 2015).

## Mitochondrial Proteins Other than MAVS as Targets of Viral Infection

Some viruses encode mitochondrial proteins, which allow them a direct functional intervention on host cells mitochondria (Table 2). In this regard, the Acanthamoeba polyphaga mimivirus (APMV), one of the largest known viruses (400 nm in its capside diameter) (La Scola et al., 2003; Monné et al., 2007), encodes a mitochondrial transport protein called VMC1 (viral mitochondrial carrier), whose function is to transport dATP and other nucleotide triphosphates (dTTP, TTP, UTP, and ADP). VMC1 can support the replication of the APMV genome by acquiring additional nucleotide triphosphates from the mitochondrial pool in exchange for cytosolic ADP (Monné et al., 2007). The APMV genome additionally encodes other five putative mitochondrial proteins (Monné et al., 2007).

**Table 2:** Viruses that target other mitochondrial proteins.

**Virus**	**Viral proteins**	**Effect**	**References**
Acantthamoeba polyphaga mimivirus (APMV)	Virus mitochondrial carrier 1 (VMC1)	Increase of viral replication by transporting dATP from the motochondrial pool	Monné et al., 2007
Epstein Barr virus (EBV)	BHRF1, BZLF1, BALF1, early Zta	Increase of viral replication, prevention of B cell apoptosis, blockage of mDNA replication	Cavallari et al., 2018
Hepatitis C virus (HCV)	p7, NS3/4A, NS5A	Disruption of mitochondrial function, cleaveage of MAVS	Cavallari et al., 2018
Hepatitis C virus (HCV)	Core	Mitochondria depolarization, increased production of mitochondrial ROS	Cavallari et al., 2018
Influenza virus (IV)	PB1-F2, PB2, NS1	Modulation of viral replication, viral mRNA synthesis	Cavallari et al., 2018
Herpes simplex virus-1 (HSV-1)	UL 12.5	Degradation of mitochondrial DNA early during infection	Cavallari et al., 2018
Herpes simplex virus-1 (HSV-1)	UL 12	Generation of mature viral genomes	Cavallari et al., 2018

The Human T-cell leukemia virus type 1 (HTLV-1) causes adult T-cell leukemia/lymphoma (ATLL) and encodes an 87-amino acid protein (p13) that helps this virus to establish a persistent infection. This protein primarily accumulates in the inner mitochondrial membrane of host cells and alters mitochondrial morphology toward a more rounded shape, fragments mitochondria (mitochondrial fission), and reduces mitochondrial Ca^2+^ uptake (Biasiotto et al., 2010; Cavallari et al., 2018).

Several proteins encoded by Epstein Barr virus (EBV) target mitochondria, such as BHRF1 (BamHI-H right reading frame), BZLF1 (also known as Zebra protein), BALF1 (BamHI-A left frame transcript), LMP2A (Latent membrane protein), and immediate early Zta protein. BHRF1 accumulates in the outer mitochondrial membrane (OMM) of B lymphocytes, preventing apoptosis and promoting survival of EBV-infected cells, viral persistence, and replication; BHRF presents homology with the transmembrane domains of some eukaryotic Bcl-2 family members (Kvansakul et al., 2017; Cavallari et al., 2018); and BZLF1 has the capacity to interact with mtSSB (mitochondrial single-stranded DNA-binding protein), which is required for the replication of the mitochondrial genome, and partially redirects mtSSB from mitochondria to the nucleus (LaJeunesse et al., 2005; Cavallari et al., 2018). BALF1 also shares homology with Bcl-2 family members and modulates apoptosis and promotes transformation (Hsu et al., 2012; Cavallari et al., 2018). LMP2A induces mitochondrial fission by a Drp1-dependent mechanism (Pal et al., 2014; Cavallari et al., 2018), and finally, the immediate early Zta protein can also bind mtSSB in the cytoplasm, inducing its re-location to the nuclei, blocking mitochondrial DNA replication and facilitating viral replication (Wiedmer et al., 2008).

Many other viruses encode mitochondrial proteins capable of regulating a broad spectrum of mitochondrial activities, as reviewed by Cavallari et al. (2018), including the control of intracellular Ca^2+^, apoptosis, mitochondrial dynamics, the levels of cytochrome c oxidase III (COXIII) and COX activity, as well as cellular ROS production, and the aggregation of mitochondria near the nucleus. Others promote mitophagy and interfere with the antiviral interferon response (Wu et al., 2007; Wang and Ryu, 2010). Proteins such as KS-Bcl-2 localize in mitochondria (Gallo et al., 2017), and others such as the KSHV-encoded K7 protein localize in mitochondria as well as in the ER and nucleus (Feng et al., 2002).

The non-structural proteins p7 of HCV can modify the mitochondrial function. The p7 protein is determinant for the assembly and later release of infectious virions, it is capable to form membrane-associated hexameric ion channels, induces mitochondrial membrane depolarization, and binds to the interferon inducible protein 6–16 (IFI6-16) (Nieva et al., 2012; Madan and Bartenschlager, 2015; Qi et al., 2017); HepG2 cells that express HCV core protein have increased levels of prohibitin, a protein that regulates mitochondrial function and apoptosis (Peng et al., 2015), by reducing the levels of COX subunits I and II. Therefore, the interaction between the HCV core protein and prohibitin may interfere with the assembly of the respiratory chain, which could lead to increased production of mitochondrial ROS and viral replication (Tsutsumi et al., 2009; Ren et al., 2016). Other molecular partners for viral-encoded mitochondrial proteins are voltage-dependent anion channel 3 (VDAC3) (Rahmani et al., 2000), and heat shock protein 60 (HSP60) (Tanaka et al., 2004).

Three influenza virus proteins are known to localize into mitochondria: PB1-F2, PB2, and NS1 (Chen et al., 2001; Yamada et al., 2004; Carr et al., 2006; Tsai et al., 2017). Although PB1-F2 is dispensable for viral replication, at least in some host cells, its expression accelerates influenza virus-induced apoptosis in human monocytes through mitochondrial ANT3 (adenine nucleotide translocator 3) and VDAC1 (voltage dependent anion channel 1) (Chen et al., 2001; Zamarin et al., 2005). The PB2 protein has a key role in viral mRNA synthesis and localizes in mitochondria, where it can regulate the viability of mitochondria during infection (Carr et al., 2006). The NS1 protein is highly expressed in Influenza A virus-infected cells, and predominantly localizes in the nucleus, although it may also be found in the cytoplasm at later stages of infection (Melén et al., 2007). Although NS1 does not harbor mitochondria-targeting sequences, it has also been found in mitochondria at early times (1.5 h) post-infection (Tsai et al., 2017).

The UL12 gene of herpes simplex virus type 1 (HSV-1) encodes two distinct but related alkaline DNases through two separately promoted 3' co-terminal mRNAs, producing full-length (UL12) and amino-terminal truncated (UL12.5) proteins. UL12 localizes to the nucleus while UL12.5 is predominantly located in mitochondria, where it degrades mitochondrial DNA early during infection. Whereas nuclear-targeted UL12 produces mature viral genomes from larger genome precursors (Draper et al., 1986; Saffran et al., 2007; Corcoran et al., 2009), the role of UL12.5 is not well-defined since mitochondrial DNA degradation is not required for HSV-1 replication (Duguay et al., 2014).

## Mitochondrial Dynamics and Viruses

Mitochondria constantly undergo fusion and fission depending on the cell metabolic requirements, a process that has been dubbed as mitochondrial dynamics (Mishra and Chan, 2016).

Along with being the “powerhouse” of eukaryotic cells, mitochondria are also involved in cellular innate antiviral immunity (Seth et al., 2005). Mitochondrial fusion and fission processes depend on the activity of mitofusin 1 (Mfn1), mitofusin 2 (Mfn2), and optic atrophy protein 1 (OPA1)—which promotes fusion—in addition to Dynamin-related protein 1 (Drp1)—which promotes mitochondrial fission (Mishra and Chan, 2016). There is evidence that antiviral immune responses can be regulated by mitochondrial dynamics (Arnoult et al., 2011; West et al., 2011). The close association between mitochondrial dynamics and several mitochondrial and cellular functions may suggest that mitochondrial dynamics could be a target for viruses to interfere with immune responses (Table 3). Likewise, the non-structural protein 4A (NS4A) from HCV, either alone or associated with the non-structural protein 3 (NS3), accumulates in mitochondria, altering the mitochondrial dynamics (Nomura-Takigawa et al., 2006). Infection with HIV-1 re-shapes mitochondrial distribution within the cells (Radovanović et al., 1999), while African swine fever virus (ASFV) induces the clustering of mitochondria around virus factories within infected cells, providing the local energy required for the release of virus (Rojo et al., 1998). The DENV NS2b3 protein partially cleaves Mfn1 and Mfn2, attenuating interferon responses (Yu et al., 2015), and induces mitochondrial fusion by inhibiting Drp1 activation and in turn the activation of the interferon response (Chatel-Chaix et al., 2016).

**Table 3:** Viruses that disrupt mitochondrial dynamics.

**Virus**	**Viral proteins**	**Effect**	**References**
Hepatitis C virus (HCV)	NS4A, NS3	Change of mitochondria distribution	Nomura-Takigawa et al., 2006
Human immunodeficiency virus-1 (HIV-1)		Clustering of mitochondria	Radovanović et al., 1999
African swine fever virus (ASFV)		Cluster of mitochondria around virus factories, providing ATP for virus release	Rojo et al., 1998
Dengue virus (DENV)	NS2b3	Cleavege of Mfn1 and Mfn2, attenuation of IFN responses	Yu et al., 2015
		Mitochondrial fusion by inhibition of Drp1	Chatel-Chaix et al., 2016
Hepatitis B virus (HBV)	HBx	Mitochondrial fission, and mitochondrial injury	Kim et al., 2013

Excessive mitochondrial fission may lead to mitochondrial damage, and this may have a role in hepatitis B virus (HBV)-induced liver disease (Kim et al., 2013).

Hepatitis B virus, through its HBx protein, triggers the translocation of Drp1 to the mitochondria by stimulating the phosphorylation of Drp1 at the Ser616 residue, and on the other hand, contributes to the degradation of Mfn2, favoring mitochondrial fission and mitophagy, attenuating the virus-induced apoptosis in the process (Kim et al., 2013).

Intracellular calcium concentrations also regulate mitochondrial dynamics since the calcium-dependent phosphatase calcineurin dephosphorylates Drp1 at S637, facilitating the recruitment of Drp1 to the mitochondria and the consequent mitochondrial fission (Cereghetti et al., 2008).

## Intracellular Calcium Homeostasis and Viral Infections

Intracellular calcium participates in cell signaling, mitochondrial function, and cell death (Duchen, 2000; Contreras et al., 2010), and Ca^2+^ uptake by mitochondria activates Krebs cycle enzymes and oxidative phosphorylation, leading to higher ATP production (Nasr et al., 2003).

Several viruses regulate host cell calcium concentrations in the cytoplasm as well as in mitochondria, allowing viral gene expression, virus replication, and the control of host cell viability (Table 4). HSV1 downregulates the uptake of Ca^2+^ by mitochondria along its lytic cycle, modulating virus replication (Lund and Ziola, 1985). Other viruses such as HCV target mitochondria, increasing Ca^2+^ concentration (Li et al., 2007; Campbell et al., 2009). Among the HCV proteins known to interfere with Ca^2+^ homeostasis, are the core protein, the NS5A, and the p7 protein (Gong et al., 2001; Griffin et al., 2004; Kalamvoki and Mavromara, 2004; Dionisio et al., 2009).

**Table 4:** Viruses that disrupt calcium homeostasis.

**Virus**	**Viral proteins**	**Effect**	**References**
Human T leukemia virus (HTLV-1)	p13	p13 accumulates in the inner mitochondrial membrane, reduces Dym and mCa^2+^ uptake	Biasiotto et al., 2010
Herpes simplex virus 1 (HSV1)	?	Modulation of viral replication by down-regulation of Ca^2+^ uptake by mitochondria	Lund and Ziola, 1985
Hepatitis C virus (HCV)	NS5A, p7	Increase of Ca^2+^ concentration	Gong et al., 2001; Griffin et al., 2004
Hepatitis B virus (HBV)	HBx	Ca^2+^ release from mitochondria and ER	Bouchard et al., 2001
Human immunodeficiency virus-1 (HIV-1)	Nef	Increase in viral replication by IP3R-dependent increase of cytosolic Ca^2+^	Foti et al., 1999
Rotavirus	NSP4	virus release by decreasing Ca^2+^ concentration	Tian et al., 1995; Ruiz et al., 2007
Poliovirus	2BC	Increase in viral gene expression and apoptosis by increse in Ca^2+^ concentration	Aldabe et al., 1997
Coxsackievirus B3	2B	Control of apoptosis and virus release by regulation of Ca^2+^ concentration	Campanella et al., 2004
Human cytomegalovirus (HCMV)	pUL37x1	Increased viral replication by mitochondria Ca^2+^ uptake and increased ATP	Sharon-Friling et al., 2006; Bozidis et al., 2010

*?, not known*.

HBV induces the mobilization of Ca^2+^ from mitochondria and endoplasmic reticulum to the cytoplasm through the interaction of the HBV protein X with voltage-dependent anion channels (VDAC) (Bouchard et al., 2001; Choi et al., 2005). The HIV-1 protein Nef (nuclear elongation factor) interacts with the Inositol 1,4,5-trisphosphate receptor (IP3Rs), increasing cytosolic Ca^2+^ concentration, promoting the transcription of virus-encoded genes and viral replication (Kinoshita et al., 1997; Foti et al., 1999). Rotavirus, through its NSP4 protein, activates phospholipase C (PLC) and the release of Ca^2+^ from the endoplasmic reticulum to the cytosol. However, by the end of its life cycle there is a decrease in cellular Ca^2+^ concentrations enabling rotavirus release (Tian et al., 1995; Ruiz et al., 2007; Díaz et al., 2008).

Poliovirus increases intracellular Ca^2+^ concentrations shortly after infection, increasing viral gene expression (Irurzun et al., 1995; Aldabe et al., 1997). By the end of the virus life cycle Ca^2+^ accumulates within mitochondria at the expense of ER stores in a mitochondrial calcium uniporter (MCU) and voltage-dependent anion channel (VDAC)-dependent process, leading to mitochondrial dysfunction and apoptosis (Brisac et al., 2010).

Enteroviruses control apoptosis through Ca^2+^ regulation; in this way, low levels of cytosolic Ca^2+^ provide the conditions for viral replication while high concentrations of cytosolic Ca^2+^ lead to the formation of vesicles which allow virus release (Campanella et al., 2004; Van Kuppeveld et al., 2005).

Human cytomegalovirus (HCMV) protein pUL37 × 1, also known as viral mitochondrion-localized inhibitor of apoptosis (vMIA) localizes into mitochondria and induces the transfer of ER Ca^2+^ into mitochondria, increasing the production of ATP and virus replication (Sharon-Friling et al., 2006; Bozidis et al., 2010).

The maturation of viral glycoproteins is dependent on both pH and intracellular Ca^2+^ concentrations. Ca^2+^ acts as a cofactor for several enzymes including glycosyl- and sulfo-transferases (Vanoevelen et al., 2007). Measles virus (MV), Dengue virus (DENV), West Nile virus (WNV), Zika virus (ZIKV), and Chikungunya virus (CHIKV) use the host calcium pump secretory pathway calcium ATPase 1 (SPCA1) for Ca^2+^ loading into the trans Golgi network, which activates glycosyl transferases and proteases allowing viral maturation and spreading (Hoffmann et al., 2017).

## mTOR and AMPK as Metabolic Hubs and Viral Targets for Evasion

The mechanistic target of rapamycin (mTOR) and the adenosine monophosphate-activated protein kinase (AMPK) constitute an integrated metabolic sensor. High levels of ATP (high ATP/AMP ratio) activate mTORC1, resulting in enhanced nutrient-dependent protein synthesis, cell growth and proliferation, whereas low levels of ATP (low ATP/AMP and ATP/ADP ratios), a hallmark of metabolic stress (starvation, hypoxia or viral infection), lead to AMPK-mediated inhibition of mTORC1 and activation of mTORC2, which restores energy homeostasis by switching the ATP-consuming biosynthetic pathways off and the ATP-producing catabolic pathways on (Hardie et al., 2012; Saxton and Sabatini, 2017).

MTOR acts as the catalytic subunit of either of two molecular complexes known as mTOR complex 1 (mTORC1) and mTOR complex 2 (mTORC2); mTORC1 is bound to the protein Raptor (Hara et al., 2002; Kim et al., 2002) and mTORC2 is bound to the protein Rictor (Hresko and Mueckler, 2005).

MTORC1 induces metabolic reprograming from OXPHOS to glycolysis by upregulating the transcription factor hypoxia-induced factor 1α (HIF1α) and, as a result, increases the expression of several glycolytic enzymes including phospho-fructo kinase (PFK). On the other hand, mTORC2 regulates cell proliferation and survival by activating the PI3K-Akt pathway (Düvel et al., 2010; Thomanetz et al., 2013; Saxton and Sabatini, 2017). The mTORC1 complex acts downstream of Akt and, as a way of regulation, the mTORC1 substrate p70S6K suppresses mTORC2, and the mTORC1 substrate Grb10 suppresses PI3K signaling (Hsu et al., 2011; Yu et al., 2011; Saxton and Sabatini, 2017), establishing a negative feedback that balances mTORC1 and mTORC2 activities (Meade et al., 2018).

Extracellular growth factors, the cell energy status, and different stressors such as viral infection are integrated into the mTOR pathway. Not surprisingly, viruses can modulate mTOR signaling to their advantage (Le Sage et al., 2016; Saxton and Sabatini, 2017) (Table 5). HSV-1 can enhance mTORC1 activity; whereas Poliovirus, HIV-1, Sindbis virus, and CHIKV can inhibit this same complex (Martin et al., 2012).

**Table 5:** Viruses that target mTOR or AMPK.

**Virus**	**Viral proteins**	**Effect**	**References**
Herpes simplex virus 1 (HSV1)	viral kinase Us3	Enhancement of mTORC1 activity	Martin et al., 2012
Poliovirus (PV)		Inhibition of mTORC1 activity	
Human immunodeficiency virus-1 (HIV-1)	Env	Activation of mTORC1 activity	Le Sage et al., 2016
Sindbis virus (SINV)		Activation of mTORC	Le Sage et al., 2016
Chikungunya virus (CHIKV)	?	Controversial activation/Inhibition of mTOR	Le Sage et al., 2016
Influenza A virus (IAV)	NS1	Differential activation of mTORC1 and mTORC2, supports viral replication	Kuss-Duerkop et al., 2017
Andes virus (ANDV)	glycoprotein Gn	Activation of mTOR, supports viral protein expression and replication	McNulty et al., 2013
Hepatitis C virus (HCV)	NS5A	Activation of mTORC1 supports viral protein expression and replication	Stohr et al., 2016
Poxviruses	F17	Evasion of cytosolic sensing by disruption of the mTORC1-mTORC2 circuit	Meade et al., 2018
Dengue virus (DENV)	?	Viral replication by activation of AMPK and inhibition of mTORC1	Jordan and Randall, 2017
Zika virus (ZIKV)	?	AMPK activation evokes antiviral innate responses and restricts virus replication	Kumar et al., 2018

*?, not known*.

Activation of mTORC1 supports viral protein expression and replication of Influenza A virus, Andes virus (ANDV), and HCV (McNulty et al., 2013; Stohr et al., 2016; Kuss-Duerkop et al., 2017). On the other hand, poxviruses are capable of evading their cytosolic sensing by means of a conserved structural protein that disrupts the mTORC1-mTORC2 regulatory circuit while maintaining the metabolic benefits of mTOR activity (Meade et al., 2018).

DENV activates AMPK, decreases the activity of mTORC1, and induces lipophagy, a process that is required for the robust DENV replication; the autophagic-mediated mobilization of lipids increases the β-oxidation in DENV-infected cells (Jordan and Randall, 2017) whereas AMPK activation evokes antiviral innate responses and restricts ZIKV replication (Kumar et al., 2018).

## Can Viruses Replicate within Mitochondria?

In addition to the interaction of viral proteins with mitochondria, which modify mitochondrial function, the Alphanodavirus flock house virus (FHV) can infect yeast, insect, plant, and mammalian cells, and replicates its RNA in the mitochondrial outer membrane. Miller et al. showed that the FHV RNA-dependent RNA polymerase, required for FHV RNA replication, localizes to the outer mitochondrial membrane and by electron microscopy these authors identified 40–60 nm membrane-bound spherical structures, similar to other virus-induced membrane structures, within the mitochondrial intermembrane space of infected cells from *Drosophila* (Miller et al., 2001).

## Concluding Remarks

This review explores how viruses may subvert immune responses by controlling host cell metabolism.

Viruses may target MAVS (RIG-I-MDA5-MAVS anti-viral pathway) interfering with RNA virus-induced type 1 interferon responses and target other mitochondrial-associated proteins, disrupting mitochondrial dynamics, mitochondrial membrane potential, and calcium handling—all of which may affect anti-viral immunity. They may also regulate the production of ATP to their advantage by interfering with mitochondrial calcium mobilization, mitochondrial enzymatic activities, and key metabolic sensors such as mTORC1, mTORC2, and AMPK. They may also induce cytotoxic T lymphocyte exhaustion, which implies metabolic reprogramming.

Viruses may also target the cGAS-STING anti-viral pathway, interfering with DNA virus-induced type I IFN responses. Since this anti-viral pathway is not directly connected with host cell metabolism (at least not in the way the RIG-I-MDA5-MAVS is), one key outstanding question is why anti-RNA viruses IFN responses are more “metabolically directed” compared to anti-DNA virus responses. Moreover, why do some RNA viruses induce the release of mitochondrial DNA and in this way recruit the RIG-I-MDA5-MAVS pathway?

In the context of HCV infection, there are at least two mechanisms accounting for the degradation of MAVS, direct cleaving by the HCV-encoded NS3/4A protein, and the NLRX1-induced proteosomal degradation. As both MAVS and NLRX1 localize in the outer mitochondrial membrane, and MAVS signaling is dependent on mitochondrial function, it remains to be determined whether NLRX1 activity is also dependent on mitochondrial function. However, it is currently known that NLRX1 regulates OXPHOS and cell integrity in a model of ischemia-reperfusion injury, and that loss of NLRX1 increases oxygen consumption and oxidative stress in epithelial cells (Stokman et al., 2017).

The role of glycolysis, β-oxidation, and oxidative phosphorylation on viral infections is continuing to emerge, but there are still outstanding questions on the role and mechanism that some metabolic intermediates may play in viral infection. For instance, dimethyl fumarate enhances the infection of cancer cell lines and human tumor biopsies with several oncolytic viruses (Selman et al., 2018), whereas ZIKV infection upregulates the enzyme cis-aconitate descarboxylase, which converts the TCA intermediate cis-aconitate to itaconate, an endogenous inhibitor of succinate dehydrogenase, inhibiting the conversion of succinate to fumarate and generating a metabolic state that restricts ZIKV replication in neurons (Daniels et al., 2019). These topics require further exploration.

On the other hand, the success of anti-viral antibody responses as well as of antibody-mediated anti-viral vaccine protection depends on plasma cell lifespan, which ultimately relies on plasma cell metabolism; something that differs from B lymphocyte metabolism (Lam et al., 2018). It would therefore be interesting to determine whether there are viruses that specifically target plasma cell metabolism, and in which case whether protecting plasma cell metabolism could be therapeutically useful in helping to support long-lasting anti-viral immune responses.

## Author Contributions

MM-A and FS-G conceived and designed the review, wrote the paper, edited, and approved the final draft. SK contributed to discussions on the paper, edited, and approved the final draft.

### Conflict of Interest Statement

The authors declare that the research was conducted in the absence of any commercial or financial relationships that could be construed as a potential conflict of interest.

## Abbreviations

ACC: Acetyil-CoA carboxylase
Akt: Akt/Protein kinase B
AMP: Adenosine monophosphate
AMPK: Adenosine monophosphate-activated protein kinase
ATP: Adenosine three phosphate
2B: 2B protein
2BC: 2BC protein
ANT3: Adenine nucleotide translocator 3
ATLL: Adult T-cell leukemia/lymphoma
BALF1: BamH1-A left frame transcript
BHRF1: BamH1-Hright reading frame
BZLF1: Zebra protein
cGAS: cyclic guanosin monophosphate-adenosin monophosphate synthase
cGMP: cyclic guanosine monophosphate
CoA: Coenzyme A
CTL: Cytotoxic T lymphocytes
COXIII: Cytochrome c oxidase III
Δψm: Mitochondrial membrane potential
Drp1: Dynamin-related protein
dTTP: Deoxythymidine triphosphate
early Zta: early Zta protein
Env: Envelope
ER: Endoplasmic reticulum
FADH2: reduced Flavin adenin dinucleotide
FAO: Fatty acid oxidation
FHV: Flock house virus
F17: F17 protein
Grb10: Growth factor receptor bound protein 10
HBx: Hepatitis B virus x protein
HIF1α: Hypoxia-induced factor 1α
HPV 18: Human papillomavirus 18
KSHV: Kaposi's sarcoma-associated herpesvirus
HSP60: Heat shock protein 60
IFI6-16: Interferon inducible protein 6-16
IFNs: Interferons
IκBα, nuclear factor of kappa light polypeptide gene enhancer in B-cells inhibitor: alpha
IKK: IκB kinase
IL-4: Interleukin-4
IP3Rs: Inositol 1,4,5-triphosphate receptors
IRF3: Interferon regulatory factor 3
ISGs: Interferon-stimulator genes
JAK-STAT: Janus kinase-Signal transductor and activator of transcription
Lag-3: Lymphocyte activation gene-3
LANA: Latency-associated nuclear antigen
LMP2A: Latent membrane protein 2A
LPS: Lypopolysacharide
M1: Macrophage type1
M2: Macrophage type 2
MAMs: Mitochondria-associated membranes
MAVS: Mitochondrial antiviral-signaling protein
MCU: Mitochondrial calcium uniporter
MDA-5: Melanoma differentiation-associated gene 5
Mfn1: Mitofusin 1
MHV68γHV68: Murine gammaherpesvirus-68
mTORC1: mechanistic target of rapamycin complex 1
mTORC2: mechanistic target of rapamycin complex 2
mtSSB: Mitochondrial single-stranded DNA binding protein
NADPH: reduced Nicotinamide adenine dinucleotide phosphate
Nef: Nuclear elongation factor
NS: Non-structural Proteins
NETs: Neutrophyl extracellular traps
NFκB: Nuclear factor kappa B
NK: Natural killer
NLR: NOD-like receptor
NOD: Nucleotide-binding and oligomerization domain
NS1: Non-structural protein 1
NS2b3: Non-structural protein 2b3
OMM: outer mitochondrial membrane
OPA1: Optic atrophy protein 1
ORF52: Open reading frame 52
OXPHOS: Oxidative phosphorylation
PB1-F2: PB1-F2 protein
PB1-F2 66S, PB1-F2 protein, serine 66 PB1-F2 66N, PB1-F2 protein: asparagine 66
PB2: PB2 protein
PD-1: Programmed death-1
PFK: Phosphofructokinase
PGC-1a: Peroxisome proliferator-activated receptor-gamma coactivator-1alpha
PI3K: phosphatidylinositol 3-kinase
PLC: Phospholipase C
PMA: Phorbol 12-myristate 13-acetate
PPP: pentose phosphate pathway
PRRs: Pattern recognition receptors
p7: protein 7
p13: protein 13
P70S6K: Ribosomal protein S6 kinase beta-1
RIG-1: Retinoic acid-induced gene 1
RLR: RIG-1-like Receptor
ROS: Reactive Oxygen Species
SPCA 1: Secretory pathway calcium ATPase 1
STING: Stimulator of interferon genes
TBK1: TANK binding kinase 1
TCA: Tricarboxylic acid
Tim-3: T cell immunoglobulin mucin-3
TLRs: Toll like receptors
TTP: thymidine triphosphate
UL 12: full length UL 12 protein
UL 12. 5: N-terminally truncated UL 12 protein
UPR: Unfolded protein response
UTP: Uridine triphosphate
VV: Vaccinia virus
VDAC3, Voltage dependent anion channel 3;VMC1: Viral mitochondrial carrier 1
vIRF1: viral Interferon regulatory factor 1
vMIA: viral mitochondrial-localized inhibitor of apoptosis. Note: Other viruses abbreviations are indicated in Tables 1–5.
